# Bioenergetic changes and mitochondrial dysfunction in mania versus euthymia in bipolar disorder type I

**DOI:** 10.1192/j.eurpsy.2023.1449

**Published:** 2023-07-19

**Authors:** A. Giménez-Palomo, M. Guitart-Mampel, G. Garrabou, X. Alsina-Restoy, A. Meseguer, L. Colomer, G. Roqué, F. J. García-García, E. Tobías, J. Moisés, M. Valentí, E. Vieta, I. Pacchiarotti

**Affiliations:** 1 Bipolar and Depressive Disorders Unit, Hospital Clínic of Barcelona, IDIBAPS; 2 Muscle Research and Mitochondrial Function Laboratory, IDIBAPS, Cellex, CIBERER, UB; 3 Pneumology Service, Hospital Clínic of Barcelona, IDIBAPS; 4 Psychology and Psychiatry Service, Fundació Clínic per a la Recerca Biomèdica, Barcelona, Spain

## Abstract

**Introduction:**

Current evidence has hypothesized the involvement of mitochondrial dysfunction during the acute episodes of BD compared to symptomatic remission. So far, no studies have compared mitochondrial and bioenergetic functions both *in-vivo* (respiratory parameters) and *ex-vivo* (cellular respiration) in different phases of the disease in the same individuals.

**Objectives:**

This multidisciplinary pilot study aims at assessing bioenergetic and mitochondrial intra-individual differences between manic and euthymic states.

**Methods:**

Four patients with a manic episode admitted to our acute psychiatric ward were recruited. Bioenergetic parameters were measured at admission (T0) and after symptomatic remission (T1).

At admission (T0) and before discharge (T1), HAMD and YMRS total scores were obtained. For the assessment of cellular respiration, polymorphonuclear cells were obtained by a Ficoll density gradient centrifugation procedure. To determine oxygen consumption (at T0 and T1), a million of living peripheral blood mononuclear cells (PBMC) were used. High-resolution respirometry was performed at 37°C by polarographic oxygen sensors in a two-chamber Oxygraph-2k system.

Specific oxygen uptakes (Routine: basal oxygen consumption; Proton Leak: oxygen consumption not coupled to ATP synthesis; and ETC: maximal capacity of the electron transport chain) rates were obtained using mitochondrial chain inhibitors and uncouplers. Oxygen consumption was normalized for protein concentration. Results are expressed as picomoles of oxygen per millilitre (pmol O2/s*μg prot).

Also, a constant work rate exercise test was performed on a cycle ergometer and basal and effort respiratory variables were measured.

Statistical analysis was performed with the SPSS v. 25.0 and GraphPad. Results were expressed as means and SD. Nonparametric tests (Mann–Whitney, Pearson) were used to determine differences (significant at p value <0.05).

**Results:**

One patient was a man and three patients were women, with a mean age of 28 years old. HAMD initial and final mean scores were 11.0 and 7.0, and mean YMRS scores were 21.5 and 7.0 respectively.

Results from mitochondrial oxygen consumption revealed that mean basal oxygen consumption tended to be higher in T1 (0.98±0.45) than in T0, and maximal respiratory capacity was significantly increased in T1 (2.26±0.33; p=0.028) compared to T0.

Mean lactate levels and pH levels were similar in T0 and T1. Scales scores were not correlated to different pH or lactate changes after the effort task. Higher initial oxygen consumption was significantly correlated to higher maximal capacity (p<0.05) in T0 and T1.

**Image:**

**Figure:**
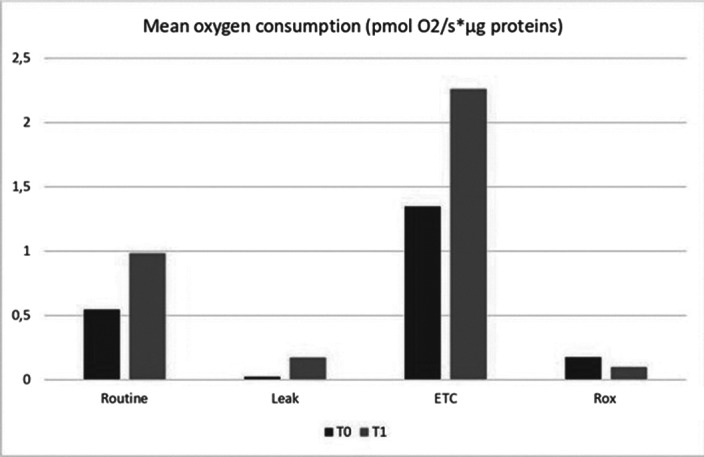

**Conclusions:**

Our preliminary results suggest that mania could imply lower oxygen consumption capacity, which should be confirmed in future studies. A bigger study is planned to determine changes in bioenergetic patterns and capacity for aerobic response in manic and depressive episodes.

**Disclosure of Interest:**

None Declared

